# The transcription factor LaMYC4 from lavender regulates volatile Terpenoid biosynthesis

**DOI:** 10.1186/s12870-022-03660-3

**Published:** 2022-06-13

**Authors:** Yanmei Dong, Wenying Zhang, Jingrui Li, Di Wang, Hongtong Bai, Hui Li, Lei Shi

**Affiliations:** 1 Key Laboratory of Plant Resources and Beijing Botanical Garden, Institute of Botany, Chinese Academy of Sciences, Xiangshan, Beijing, 100093 China; 2 University of Chinese Academy of Sciences, Beijing, 100015 China

**Keywords:** *Lavandula angustifolia*, Molecular cloning, bHLH transcription factors, Stress-responsive, Terpenoid biosynthesis

## Abstract

**Background:**

The basic helix-loop-helix (bHLH) transcription factors (TFs), as one of the largest families of TFs, are essential regulators of plant terpenoid biosynthesis and response to stresses. Lavender has more than 75 volatile terpenoids, yet few TFs have been identified to be involved in the terpenoid biosynthesis.

**Results:**

Based on RNA-Seq, reverse transcription-quantitative polymerase chain reaction, and transgenic technology, this study characterized the stress-responsive transcription factor LaMYC4 regulates terpenoid biosynthesis. Methyl jasmonate (MeJA) treatment increased volatile terpenoid emission, and the differentially expressed gene *LaMYC4* was isolated. *LaMYC4* expression level was higher in leaf than in other tissues. The expression of *LaMYC4* decreased during flower development. The promoter of *LaMYC4* contained hormone and stress-responsive regulatory elements and was responsive to various treatments, including UV, MeJA treatment, drought, low temperature, *Pseudomonas syringae* infection, and NaCl treatment. *LaMYC4* overexpression increased the levels of sesquiterpenoids, including caryophyllenes, in *Arabidopsis* and tobacco plants. Furthermore, the expression of crucial node genes involved in terpenoid biosynthesis and glandular trichome number and size increased in transgenic tobacco.

**Conclusions:**

We have shown that the stress-responsive MYC TF LaMYC4 from ‘Jingxun 2’ lavender regulates volatile terpenoid synthesis. This study is the first to describe the cloning of *LaMYC4*, and the results help understand the role of LaMYC4 in terpenoid biosynthesis.

**Supplementary Information:**

The online version contains supplementary material available at 10.1186/s12870-022-03660-3.

## Background

Volatile terpenoids are the most abundant class of volatile metabolites in plants and are involved in defense responses. Plants are exposed to environmental stresses, including abiotic (such as salt and drought) and biotic (such as pathogens and herbivores) stresses [1, 2], and adopt multiple defense mechanisms against stresses for growth and survival [3]. Volatile terpenoids protect plants against herbivores [4, 5] and thermal and oxidative stress [6] and mediate chemical communication [7, 8]. Moreover, plants synthesize monoterpenoids and sesquiterpenoids [9–13]. Among them, (−)-thujopsene and β-caryophyllene promote lateral root formation and induce plant resistance to microbes [9, 14, 15]. The sesquiterpenoid β-caryophyllene binds to the transcriptional co-repressor TOPLESS complex and modulates jasmonic acid (JA)-mediated signalling [16]. And caryophyllene induces defense responses via JA signalling [17].

Terpenoid biosynthesis begins with the formation of isopentenyl diphosphate (IPP) and its allylic isomer, dimethylallyl diphosphate (DMAPP), through the mevalonate pathway in the cytosol and the 2-C-methyl-D-erythritol 4-phosphate (MEP) pathway in plastids [18]. The enzymes 3-hydroxy-3-methyl glutaryl coenzyme A reductase (HMGR), 1-deoxyxylulose- 5-phosphate synthase (DXS), and deoxyxylulose 5-phosphate reductoisomerase (DXR) control terpenoid synthesis [19, 20]. Most of the monoterpenes are derived from geranyl diphosphate (GPP; C10) or neryl pyrophosphate (NPP), which is synthesized in a head-to-tail condensation reaction of isopentenyl diphosphate (IPP) and dimethylallyl diphosphate (DMAPP) by GPP synthases (GPPS) or NPP synthases (NPPS). Then, farnesyl pyrophosphonate synthase (FPPS) adds two IPP molecules to DMAPP to form the C15 diphosphate precursor of sesquiterpenes [21].

Terpenoid biosynthesis is regulated by structural genes and transcription factors (TFs). TFs modulate gene expression by changing transcription rates [22, 23]. Basic helix-loop-helix (bHLH) TFs play a pivotal role in plant growth and development, stress response, and the biosynthesis of secondary metabolites [24]. MYC family members are bHLH TFs [25]. Some MYC TFs control terpenoid biosynthesis in plants [26]. For instance, CpMYC2 and AtMYC2 regulate caryophyllene synthesis in *Arabidopsis thaliana* [27, 28], and SlMYC1 controls terpenoid emission in tomato (*Solanum lycopersicum*) [29]. MYC TFs have been characterized in *A. thaliana* [27, 30], *S. lycopersicum* [29], *Artemisia annua* [31], and other plants [28, 32, 33], but not in lavender.

Lavender is a model for studying the regulation of terpenoid synthesis [34]. More than 75 volatile terpenoids were identified in *Lavandula angustifolia* [35, 36]. One hundred terpene synthases (TPSs) have been identified in lavender, of which 11 were characterized, and some are induced by methyl jasmonate (MeJA) [13, 37]. Recently, a reference genome for the ‘Jingxun 2’ lavender cultivar was created [37].

This study isolated the MYC TF LaMYC4, which regulates terpenoid biosynthesis. The expression of *LaMYC4* was upregulated by UV, low temperature, drought, MeJA, and *Pseudomonas syringae* infection. Moreover, LaMYC4 overexpression increased the levels of terpenoids (especially caryophyllene) and the number and size of glandular trichomes (GTs) in transgenic plants. These results demonstrate that LaMYC4 can be a candidate gene for *L. angustifolia* molecular breeding.

## Results

### MeJA affects volatile terpenoid biosynthesis

**Fig. 1:**
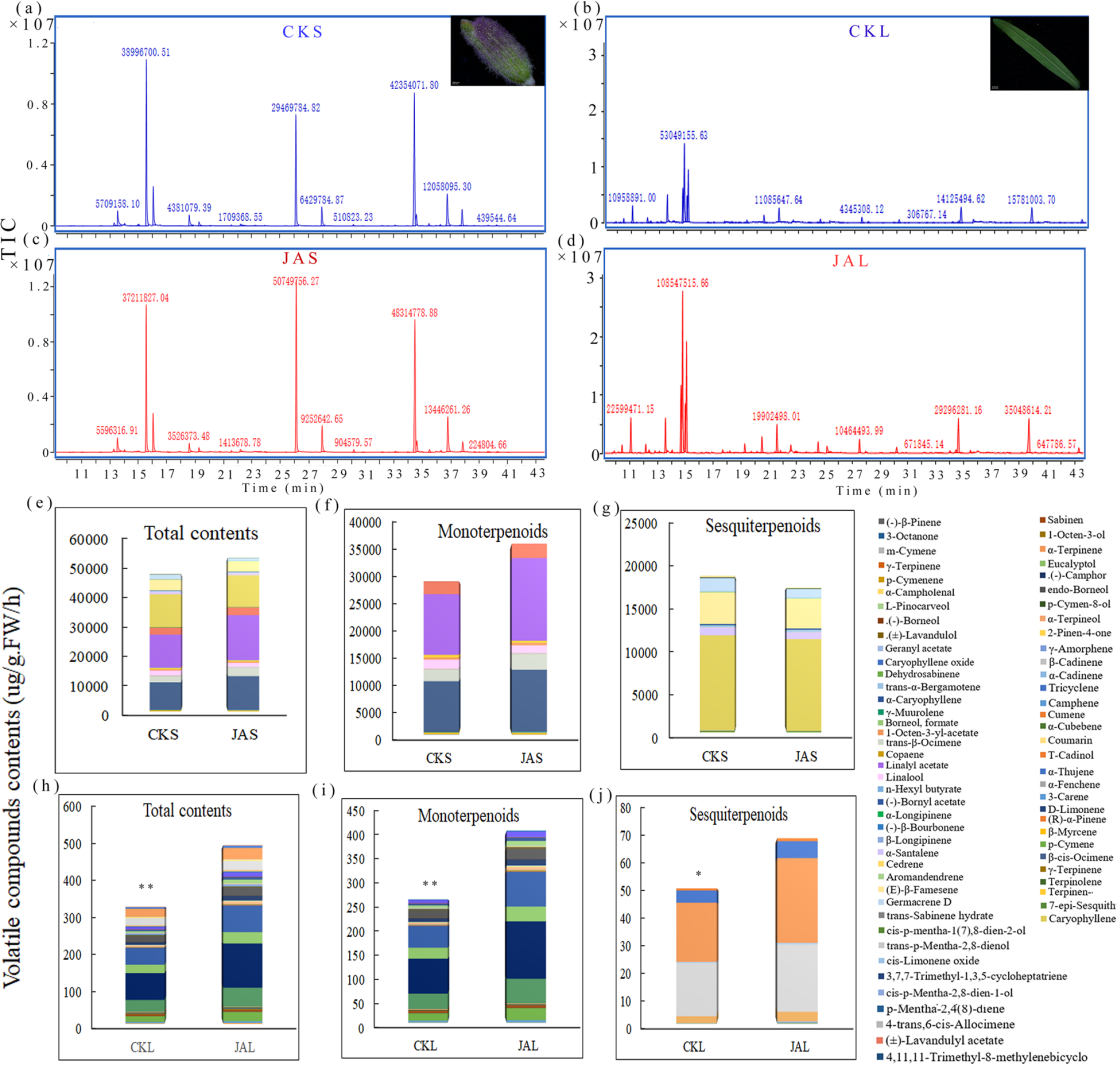
Emission of volatile terpenoids in lavender with 8 mM MeJA treatment. (**a**, **c**) GC-MS/MS chromatograms from the lavender sepal. (**b**, **d**) GC-MS/MS chromatograms from the lavender leaf. (**e**, **f**, **g**) Terpenoid contents from the lavender sepal (control, CKS, treatment, JAS). (**h**, **i**, **j**) Terpenoid contents from lavender leaf (control, CKL, treatment, JAL). The number on the peak represents the peak area. Values shown are mean ± SD at least three replicates, and standard errors are indicated as vertical lines on the top of each bar, *p < 0.05; **p < 0.01; ***p < 0.001; *t*-test

Lavender plants were treated with or without 8 mM of MeJA, and volatile terpenoids were analyzed by solid-phase microextraction gas chromatography/mass spectrometry (SPME-GC-MS). The results revealed that MeJA induced various volatile terpenoid emission, and production was significantly higher in leaf (Fig. 1 and Additional file 10: Table S1). Furthermore, MeJA promoted the emission of β-myrcene, β-cis-ocimene, and caryophyllene in lavender sepal and leaf (JAS and JAL) (Additional file 1: Fig. S1).

### Isolation and bioinformatics analysis of LaMYC4

**Fig. 2:**
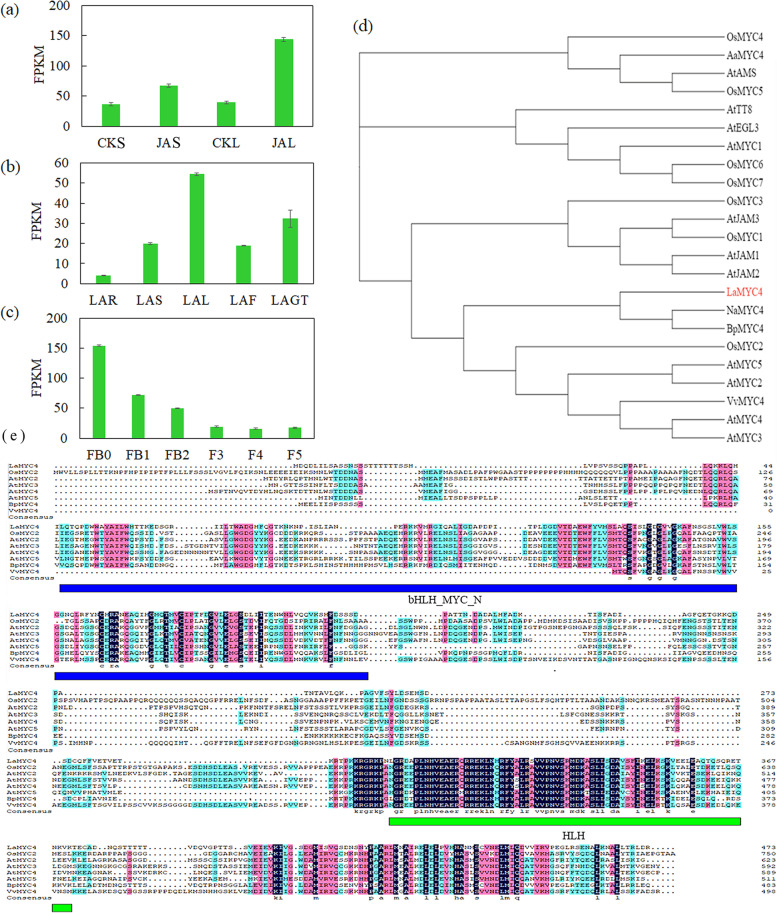
Characterization of LaMYC4. (**a**) Transcriptional changes in response to MeJA. (**b**) The expression levels of *LaMYC4* in different tissues of *L. angustifolia* (LAR, root; LAS, stem; LAL, leaf; LAF, flower; LAGT, glandular trichome). (**c**) The expression levels of *LaMYC4* during flower development. (**d**) Phylogenic tree analysis of LaMYC4 and MYC TFs from *Arabidopsis thaliana*, *Artemisia annua*, *Oryza sativa*, etc. The phylogenic tree was constructed on MEGA7.0 by using the neighbor-joining method, and the bootstrap values were obtained for 1000 replications. (**e**) Multiple alignments of LaMYC4 with related MYC proteins from other plant species. Values shown are mean ± SD of three replicates, and standard errors are indicated as vertical lines on the top of each bar

Twenty-six MYCs were previously identified (unpublished) in *L. angustifolia* based on genome data (PRJNA642976), and the MYC gene *LaMYC4* was differentially expressed by MeJA treatment (Fig. 2a). The level of *LaMYC4* expression was significantly higher in leaf than in other tissues and decreased during flower development (Fig. 2b, c). The 1422-bp open reading frame of *LaMYC4* encoded 473 amino acids (Additional file 2: Fig. S2). Bioinformatics analysis indicated that the LaMYC4 protein contained a bHLH-MYC sequence between amino acids 38 and 211, corresponding to the N-terminal region of MYB and MYC TFs, and DNA-binding domains between amino acids 299 and 373 (Fig. 2e). Physicochemical characterization using ExPASy showed that LaMYC4 had a molecular mass of 52.24 kDa and an isoelectric point of 5.75. LaMYC4 protein was clustered into subfamily 2 or subgroup-III(d + e) according to the classification and nomenclature of AtbHLH proteins (Additional file 3: Fig. S3). A phylogenetic tree was constructed with LaMYC4 and 22 MYCs from different plants (Additional file 11: Table S2) and showed that LaMYC4 was most closely related to NaMYC4 and BpMYC4 (Fig. 2d).

### Analysis of the *LaMYC4* promoter sequence and response to stresses

**Fig. 3:**
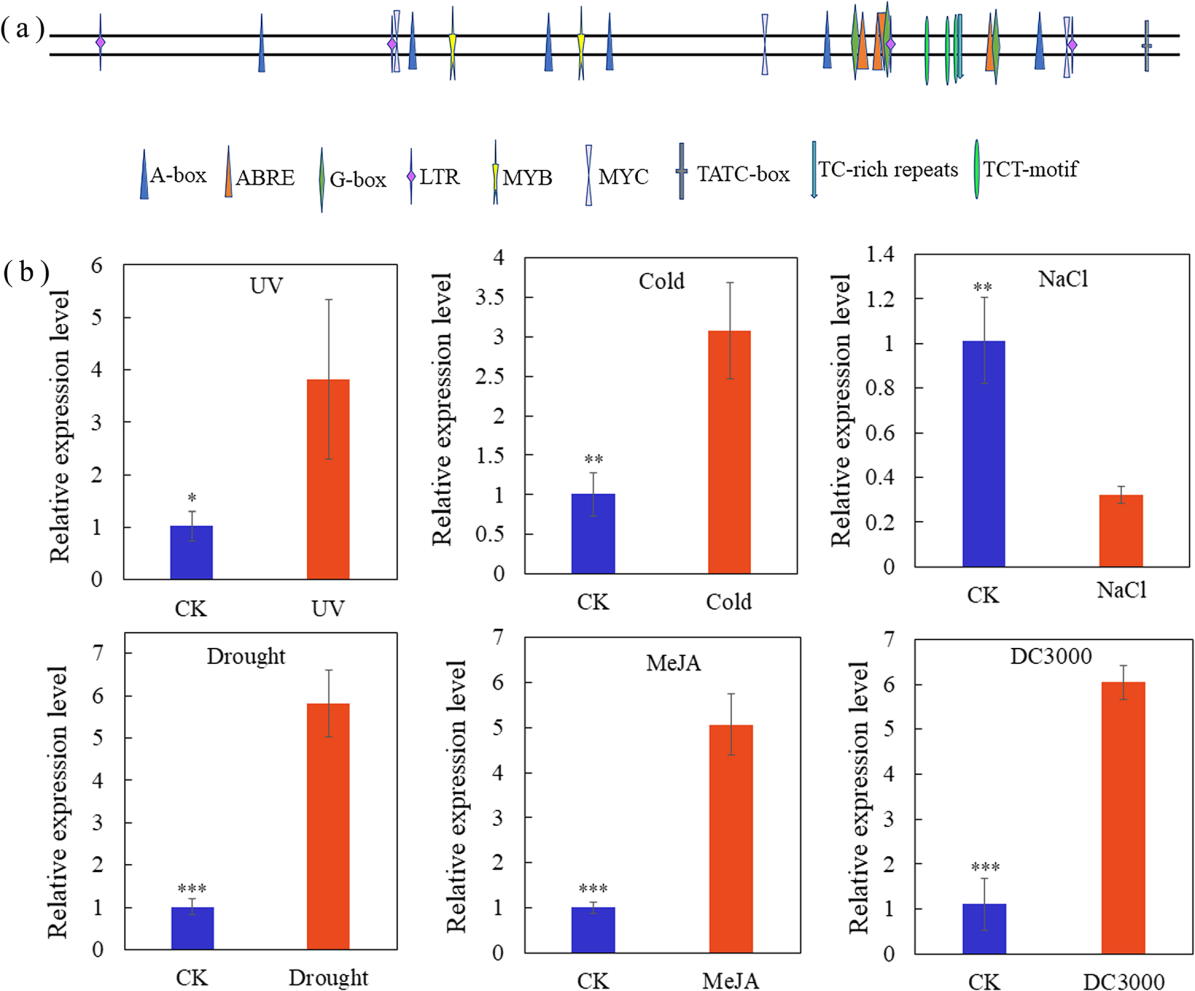
Analysis of *LaMYC4* promoter sequence and transcriptional abundance of *LaMYC4* under different stress conditions in lavender leaf. (**a**) Putative cis-acting regulatory elements were identified in the promoter sequence of *LaMYC4* using the PlantCARE database. (**b**) Treatments included UV, cold, NaCl, drought, MeJA and *Pst* DC3000. The relative expression of *LaMYC4* was quantified by qRT-PCR. Values shown are the means ± SD at least three replicates, and standard errors are indicated as vertical lines on the top of each bar. *p < 0.05; **p < 0.01; ***p < 0.001; *t*-test

The 2000-bp promoter upstream of the 5′-untranslated region (5′ UTR) was analyzed using PlantCARE software (Additional file 12: Table S3). Four abscisic acid response elements were found at + 1432, − 1469, − 1467, and + 1687 bp, three light or abscisic acid response elements (G-box) were located at − 1431, + 1469, and − 1686 bp, four low-temperature response elements were situated at − 104, − 1478, + 614, and − 1809 bp, one TC-rich repeat element involved in defense and stress response was located at − 1617 bp, and one gibberellin response element (TATC-box) was located at − 1953 bp (Fig. 3a and Additional file 12: Table S3).

Gene expression studies have shown that MYC transcription increased in response to biotic and abiotic stresses. *LaMYC4* expression levels were quantified by reverse transcription-quantitative polymerase chain reaction (RT-qPCR). The results showed that *LaMYC4* expression was significantly upregulated in lavender leaf by UV (~ 4-fold), cold (~ 3-fold), drought (~ 6-fold), MeJA (~ 5-fold), and *Pseudomonas syringae* pv. tomato (*Pst*) DC3000 (~ 6-fold) and downregulated 3-fold by NaCl (Fig. 3b).

### Subcellular localization and transactivation activity of LaMYC4

**Fig. 4:**
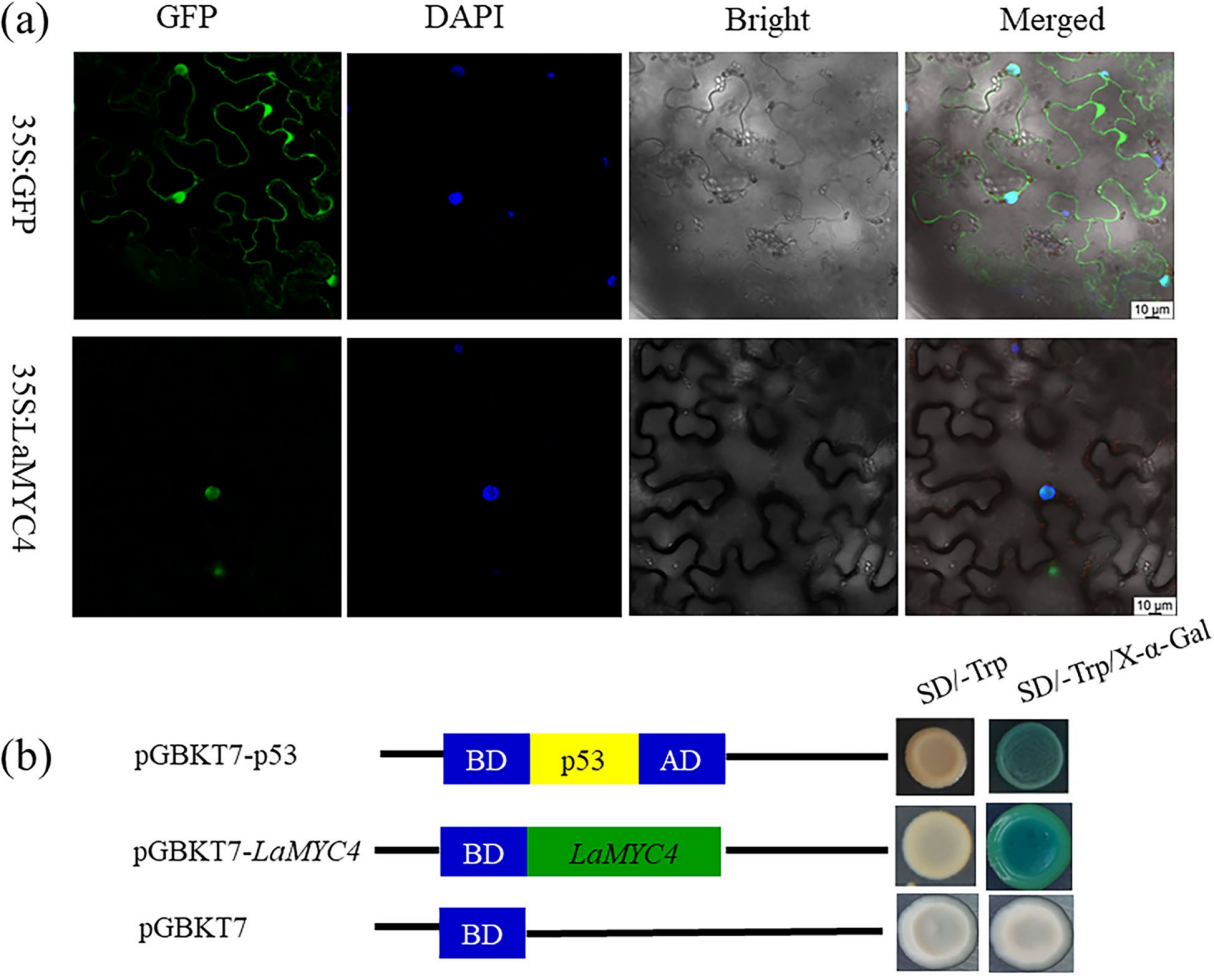
Subcellular localization of LaMYC4 in *N. benthamiana* and transcriptional activity of LaMYC4 in a yeast assay system. (**a**) Tobacco leaves were transformed with constructs including control (GFP) or fusion plasmids (LaMYC4::GFP) and 10 μg / mL DAPI was then used to stain the tobacco leaf before visualization.  (**b**) Yeast cells (strain AH109) transformed with the positive control vector (top panel), the fusion vector-containing LaMYC4 (middle panels) and the negative control vector (bottom panels)

The subcellular localization of the LaMYC4 protein was assessed using a transient expression assay in tobacco (*Nicotiana benthamiana*) leaf. The results showed that 35S::GFP was found in the cytoplasm and nucleus of plant cells, whereas LaMYC4 fusion proteins were only present in the nucleus (Fig. 4a), suggesting that LaMYC4 localizes to the nucleus.

The yeast strain AH109 and the pGBKT7 vector containing the DNA-binding domain of GAL4 were used to measure the transactivation activity of LaMYC4. Yeast cells transformed with any vector were cultivated in SD/−Trp medium. Yeast cells transformed with the fusion plasmid (pGBKT7-LaMYC4) or positive control plasmid (pGBKT7-p53) and cultivated in SD/−Trp/X-α-Gal medium appeared blue, whereas yeast cells transformed with the negative control plasmid pGBKT7 did not turn blue (Fig. 4b), indicating that LaMYC4 has transactivation activity in yeast.

### *LaMYC4* overexpression increases sesquiterpenoid biosynthesis in *A. thaliana*

**Fig. 5:**
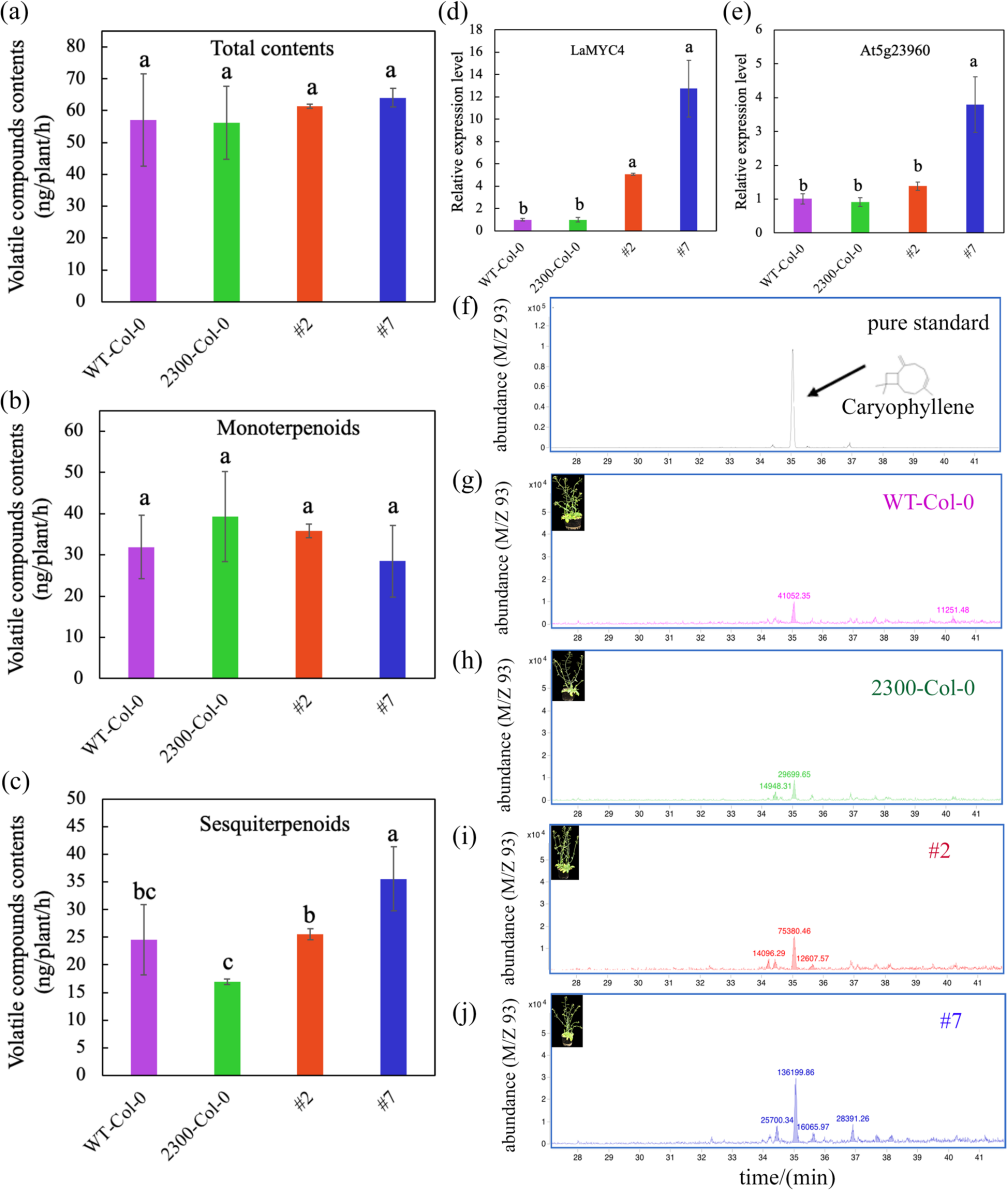
Analysis of overexpressed *LaMYC4* gene in *Arabidopsis* plants. Wild type (WT), transformed by the empty vector pCAMBIA2300S (2300) and overexpressed *LaMYC4* gene (35S::LaMYC4) plants (#2, #7). (**a**) Total contents. (**b**) monoterpenoids. (**c**) sesquiterpenoids. (**d**) Relative expression level of *LaMYC4* as verified by qRT-PCR. (**e**) Relative expression level of *At5g23960* as verified by qRT-PCR. (f-j) GC trace of caryophyllene. The number on the peak represents the peak area. The products were identified by comparison with compounds in the library NIST14 and reference standards. The values shown are mean ± SD at least three replicates. Standard errors are indicated as vertical lines on the top of each bar, and bars annotated with different letters were significantly different according to Fisher’s LSD test (*P* < 0.05) after ANOVA

Under the control of the CaMV 35S promoter, *LaMYC4* was overexpressed in transgenic *A. thaliana* by *Agrobacterium tumefaciens*-mediated transformation. Terpenoid levels were measured in transgenic plants from the T3 generation. The results indicated that the expression of LaMYC4 was significantly changed in transgenic lines, while the contents of total terpenoids and monoterpenoids did not change significantly (Fig. 5a, b, e). In contrast, sesquiterpenoid levels increased 0.5–1.0-fold in transgenic lines overexpressing *LaMYC4* (#2, #7) compared with the empty vector group (Fig. 5c). In addition, caryophyllene was the most abundant sesquiterpenoid in *A. thaliana*, and its emission was more than 2-fold higher in transgenic *A. thaliana* than the control groups (wild-type and empty vector plants) (Fig. 5d and Additional file 4: Fig. S4). The expression of caryophyllene synthase (*At5g23960*) in transgenic *A. thaliana* (#7) was also significantly increased (Fig. 5f).

### Overexpression of *LaMYC4* increases volatile terpenoid biosynthesis in tobacco

**Fig. 6:**
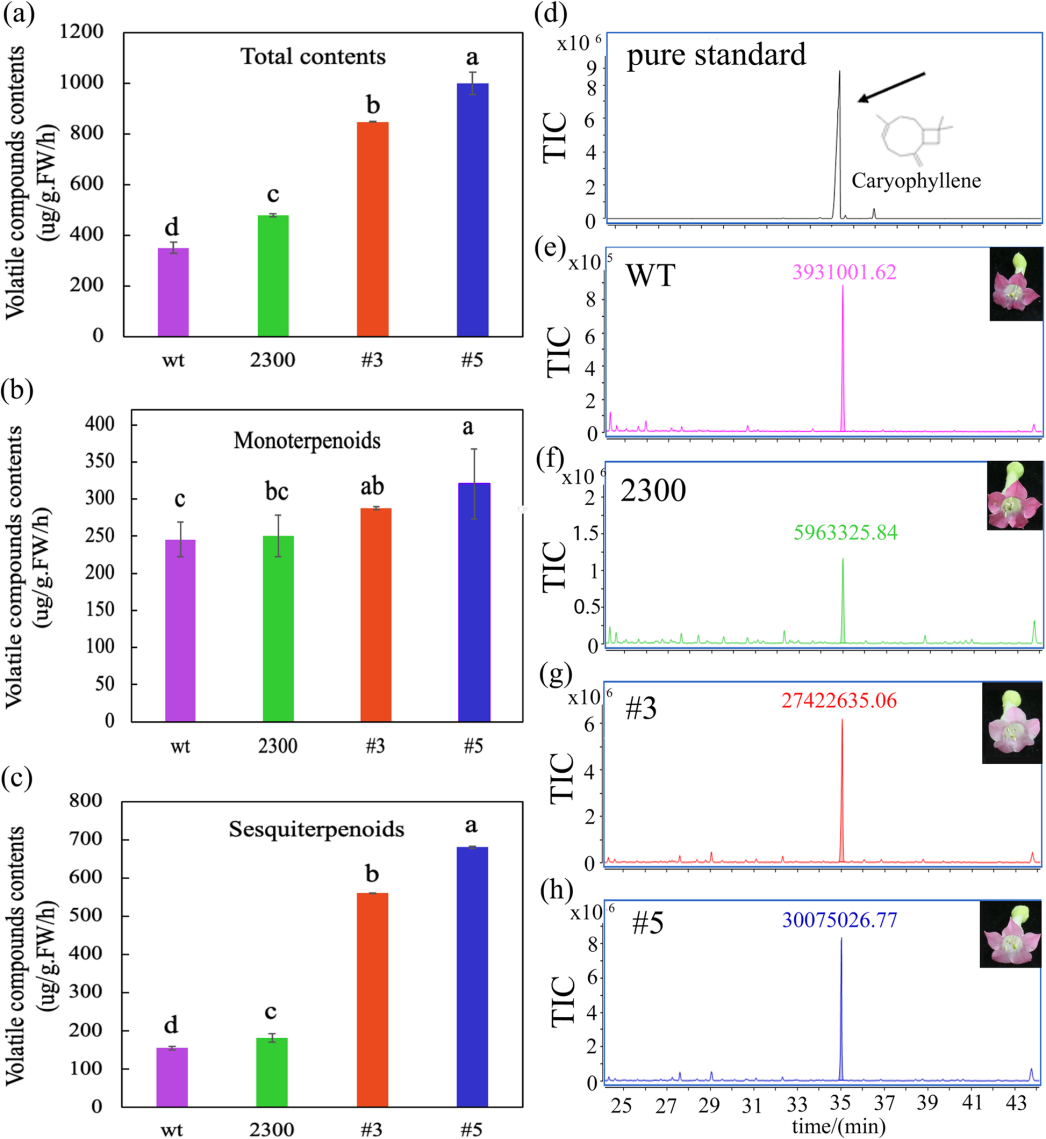
SPME-GC-MS analysis of VOCs from the tobacco floral. Wild type (WT), transformed by the empty vector pCAMBIA2300S (2300) and overexpressed *LaMYC4* gene (35S::LaMYC4) plants (#3, #5). (**a**) Total contents. (**b**) monoterpenoids. (**c**) sesquiterpenoids. (d-h) GC trace of caryophyllene. The number on the peak represents the peak area. The products were identified by comparison with compounds in the library NIST14 and reference standards. The values shown are mean ± SD at least three replicates. Standard errors are indicated as vertical lines on the top of each bar, and bars annotated with different letters were significantly different according to Fisher’s LSD test (*P* < 0.05) after ANOVA

Under the CaMV 35S promoter, *LaMYC4* was overexpressed in tobacco by *Agrobacterium tumefaciens*-mediated transformation. Terpenoid concentrations were quantified in transgenic plants from the T2 generation using SPME-GC-MS. The results indicated that total volatiles and sesquiterpenoid contents increased 1–2-fold and 2–3-fold in transgenic tobacco, respectively, compared with the control (Fig. 6a, c), whereas monoterpenoid contents increased significantly only in transgenic line #5 (Fig. 6b). The contents of phytohormones Zr, IAA, JAs decreased in transgenic tobacco compared with the control, while the contents GA3 and ABA increased, and all changes were significant in transgenic line #5 (Additional file 6: Fig. S6). Caryophyllene contents were higher in lines #3 and #5 than in control plants (Fig. 6 d). Caryophyllene levels were ~ 5-fold higher in transgenic lines overexpressing *LaMYC4* (#3 and #5) than in empty vector plants (Additional file 5: Fig. S5). Furthermore, transgenic tobacco plants (35S:: LaMYC4) showed reduced flower color and increased plant height (Additional file 7: Fig. S7) compared with control plants.

### *LaMYC4* overexpression upregulates genes related to terpenoid synthesis in tobacco

**Fig. 7:**
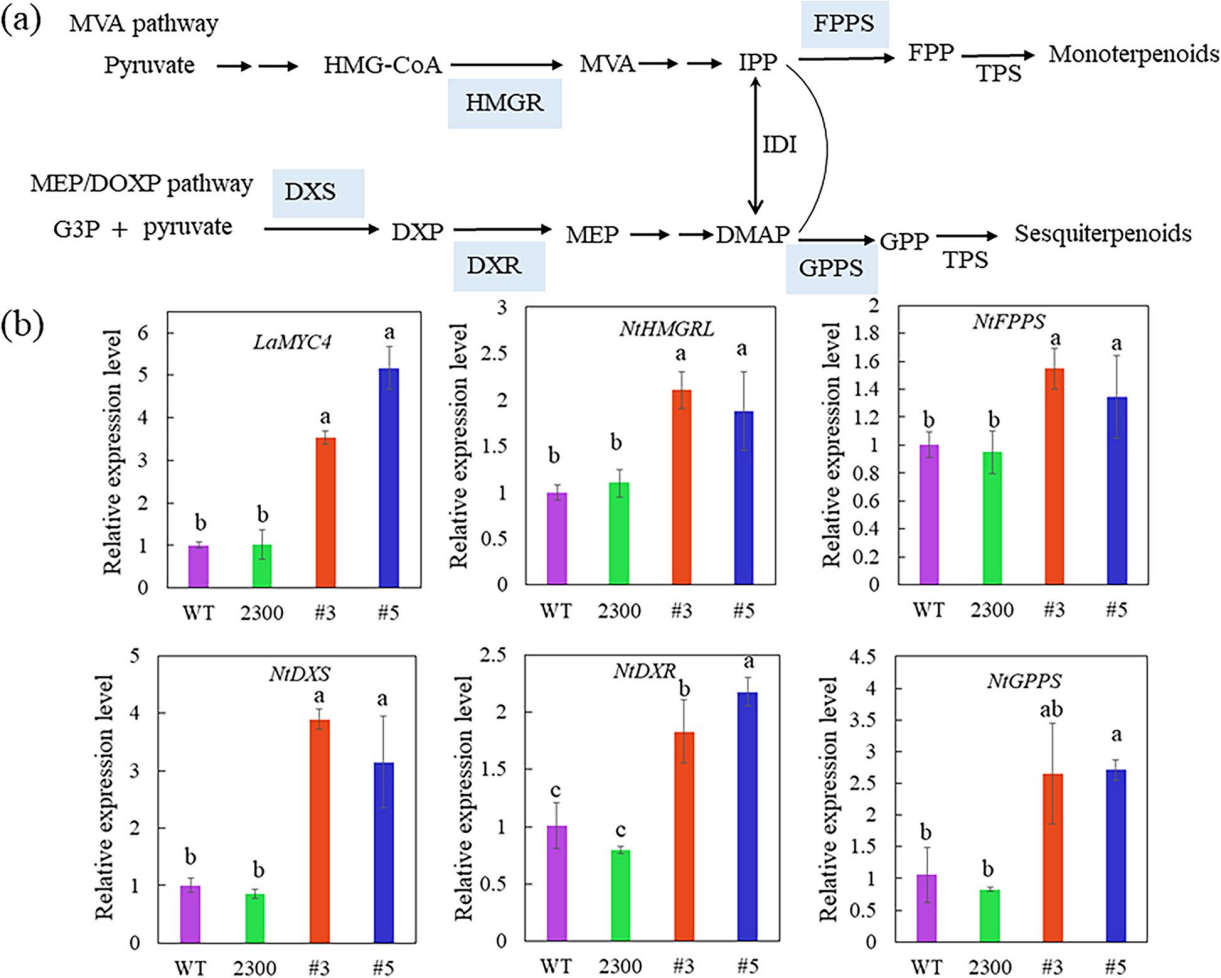
Transcript levels of *LaMYC4* and genes related to terpenoid synthesis in the tobacco floral (**a**) Schematic representation of terpenoid synthesis. (**b**) Relative expression levels of these genes related to terpenoid synthesis were determined. The values shown are mean ± SD at least three replicates. Standard errors are indicated as vertical lines on the top of each bar, and bars annotated with different letters were significantly different according to Fisher’s LSD test (*P* < 0.05) after ANOVA

To assess the effect of LaMYC4 on the expression of genes related to terpene synthesis, we investigated HMGR, FPPS, DXS, DXR, and GPPS (the sequences are shown in Additional file 13: Table S4), which are key enzymes in the MVA and MEP pathways. The expression of genes *HMGR*, *FPPS*, *DXS*, *DXR*, and *GPPS* increased 1.3-to 3.8-fold (Fig. 7b) in *LaMYC4*-overexpressing transgenic tobacco flower. In addition, *DXR* expression was strongly associated with the expression of *LaMYC4*. These results indicate that LaMYC4 was involved in the regulation of terpenoids and affects the expression of several key genes (*HMGR*, *FPPS*, *DXS*, *DXR*, and *GPPS*) in terpenoid synthesis pathway. In addition, we found that the expression of diterpenoid-related synthase (*NtCPS2* and *NtABS*) in transgenic tobacco (#5) was significantly decreased, while the expression of *NtCBTS* was significantly increased in transgenic tobacco (#3 and #5) (Additional file 8: Fig. S8).

### *LaMYC4* overexpression increases the number and size of GTs

**Fig. 8:**
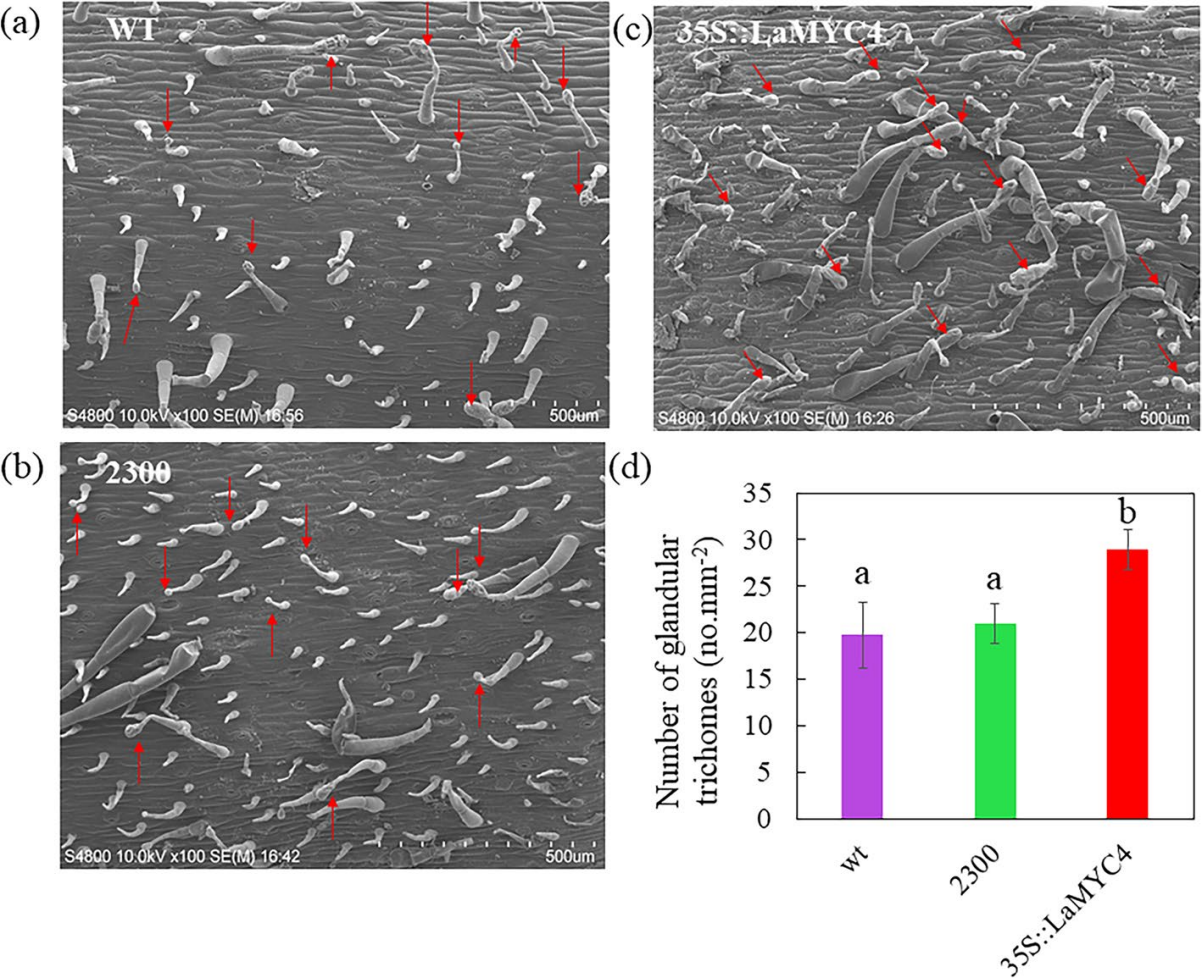
Morphology and number of glandular trichomes on tobacco stems. (**a-c**) Glandular trichomes of wild-type (WT), empty vector pCAMBIA2300 (2300) and overexpression of *LaMYC4* transgenic plants (35S::LaMYC4) on stem surfaces. (**d**) Number of glandular trichomes on the stem surfaces of wild-type (WT), empty vector pCAMBIA2300 (2300) and overexpression of *LaMYC4* transgenic plants (35S::LaMYC4). The type of trichome was glandular trichomes. A total of five plants were selected, and three completely randomized fields of view were chosen for examination. Red arrows indicate glandular trichomes. The bars represent the mean values (± SD), calculated from three to four scanning-electron micrographs of stems from different plants. Standard errors are indicated as vertical lines on the top of each bar, and bars annotated with different letters were significantly different according to Fisher’s LSD test (*P* < 0.05) after ANOVA

GTs are a physical defense to insect herbivores in response to mechanical stimulation. Moreover, evidence indicates that glandular secretory trichomes (GSTs) synthesize and store terpenoids. Since LaMYC4 regulates terpenoid biosynthesis in transgenic lines, we examined GT morphology by scanning electron microscopy. GTs on the stems of the fourth fully grown internode of 35S::LaMYC4 tobacco plants had longer stalks and larger glandular heads than control plants (Fig. 8). Moreover, the number of GTs was 0.4-fold higher in 35S::LaMYC4 tobacco plants than in control plants (Fig. 8d).

## Discussion

Plants utilize various physiological and biochemical processes to survive and respond to stresses [38, 39]. Plant bHLH proteins play a pivotal role in stress responses. For instance, OsbHLH148 and OsbHLH006 (RERJ1) respond to drought stress through the JA signalling pathway [40, 41]. *Vitis vinifera* bHLH1 responds to drought and salinity via the accumulation of flavonoids and is the regulation of abscisic acid (ABA) synthesis [42]. RsICE1 interacts with CBF/DREB1 in rice plants to improve cold tolerance [43]. We identified the promoter region of LaMYC4 by genomic analysis [37]. This region contains stress-related cis-elements that allow LaMYC4-encoded TFs to adapt to the environment. As shown in Table S5 (Additional file 14), most of the proteins of subfamilies 1, 2, 4, 10, 13, 14 and 18 responded to different biotic and abiotic stresses, such as drought, cold and salt [44]. In additional, the results of UV, MeJA treatment, drought, low temperature, *Pseudomonas syringae* infection, and NaCl treatment indicated that LaMYC4 responded to multiple stresses.

Plant bHLH TFs play vital roles in terpenoid biosynthesis. For instance, AtMYC2 binds to the promoter of the caryophyllene biosynthetic pathway genes *TPS21* and *TPS11* and stimulates gene expression [27], and CrBIS2 plays an essential role in the generation of monoterpenoid indole alkaloids [45]. LaMYC4 overexpression enhanced terpenoid synthesis, especially sesquiterpenoid caryophyllene (Additional files 4, 5: Fig. S4 and 5). TFs can simultaneously participate in the expression regulation of multiple key genes in terpenoid synthesis [46]. The transcript levels of the structural genes *HMGR*, *FPPS*, *DXR*, *DXS*, *GPPS* from the terpenoid biosynthesis pathway were significantly increased in *LaMYC4*-overexpressing lines (Fig. 7). However, the increase of monoterpenoids was not as significant as that of sesquiterpenoids. Previous studies have shown that the expression of monoterpene synthase *At1g61680* and sesquiterpene synthases *At5g23960* and *At5g44630* was increased in *CpMYC2*-overexpressing *Arabidopsis*, while the expression of monoterpene synthase *At3g25810* was decreased [28]. *LaMYC4* overexpression enhanced the flux of terpenoid biosynthetic pathways, and the decrease in anthocyanin accumulation in transgenic plants produced light-colored flowers (Additional file 7: Fig. S7). Anthocyanin production is metabolically expensive, and the overexpression of *VvmybA1* resulted in the accumulation of anthocyanins in leaf, whereas the concentration of most volatile compounds decreased in the leaf of transgenic plants [47]. The overexpression of *CpbHLH13* increased the concentration of volatile terpenoids and decreased anthocyanin accumulation [28]. These results indicated that LaMYC4 modulated volatile terpenoid biosynthesis, especially sesquiterpenoid caryophyllene, and influenced carbon flow in the terpenoid pathway.

MYC3 and MYC4 activate JA-regulated responses and act synergistically with MYC2 to control different subsets of JA-dependent transcriptional activity [48]. Different volatile compounds are involved in JA-associated stress response [49–52]. MeJA treatment confirmed the result in our study. And this study found that *LaMYC4* overexpression in tobacco increased ABA and GA3 contents and decreased JA and IAA levels (Additional file 6: Fig. S6). Abe et al. [30] have shown that AtMYC2 acts as a transcriptional activator in ABA signalling in *Arabidopsis*. Moreover, GA is involved in cell elongation [53]. Plant height increased in *LaMYC4*-overexpressing tobacco (Additional file 7: Fig. S7a, b). The morphology of stem epidermal cells was examined by scanning electron microscopy. The length of these cells was 0.3-fold higher in transgenic plants than in control plants (Additional file 7: Fig. S7). These results indicate that LaMYC4 promotes the elongation of epidermal cells by upregulating GA, increasing plant height.

Trichomes serve as physical barriers to insect herbivores [29]. Evidence indicates that GSTs produce and accumulate terpenoids [54]. In tomato, SlMYC1 regulates GT formation and terpenoid biosynthesis [29]. *LaMYC4*-overexpression in tobacco confirmed the results that MYC plays a pivotal role in plant GT formation and terpenoid biosynthesis. In addition, the increase in terpenoid levels was significantly higher in *LaMYC4*-overexpressing tobacco than in *LaMYC4*-overexpressing *A. thaliana*, which may be because there is a lack of GTs in *A. thaliana*. In conclusion, we have shown that the stress-responsive MYC TF LaMYC4 from ‘Jingxun 2’ lavender regulates terpenoid synthesis. *LaMYC4*-overexpressing plants accumulated more terpenoids, especially sesquiterpenoid caryophyllene. In addition, LaMYC4 may be involved in regulating GT formation, increasing terpenoid biosynthesis and accumulation.

## Conclusions

This study provides, to our knowledge, the first to describe the cloning of *LaMYC4*. We successfully profiled the tissue-specific expression patterns based on RNA-Seq. Different stress treatments and analysis of the *LaMYC4* promoter sequence shown that *LaMYC4* responds to multiple stress to adapt to the environment. Furthermore, *LaMYC4-*overexpression increased the levels of terpenoids (especially caryophyllene) and the number and size of GTs in transgenic plants. These results demonstrate that *LaMYC4* can be a candidate gene for *L. angustifolia* molecular breeding. And our study served as a basis for future studies on the regulation of terpenoid synthesis and stress responses by MYCs.

## Methods

### Plant materials and treatments

The *L. angustifolia* cultivar used in this study was ‘Jingxun 2’ from the Institute of Botany, Chinese Academy of Sciences. The voucher specimen of ‘Jingxun 2’ was kept at the Chinese national herbarium, Institute of Botany, Chinese academy of sciences (voucher specimen: 02308796). All wild-type *Arabidopsis* and tobacco seeds used were obtained from Key Laboratory of Plant Resources. And all plant material was used in accordance with relevant guidelines and regulations. Transcriptome data were obtained from a previous study [13, 37]. For *Pst* DC3000, UV, MeJA, salinity (NaCl), cold, and drought treatments, 12 one-year-old potted plants of the same cultivar (for each treatment) were grown in a greenhouse. *Pst* DC3000 inoculation was performed for 6 h as described previously [55]. UV treatment lasted 10 minutes a day for 3 days. MeJA treatment was with 8 mM for 12 h. NaCl treatment with 300 mM was once every 3 days, twice in total, sampling on the seventh day, and watering thoroughly each time. Cold (16 °C) and or drought treatments were for 7 days. Sepal, leaf and flower were removed from potted plants for further analysis. *L. angustifolia*, *A. thaliana* (Col-0), and tobacco (*Nicotiana benthamiana* and *N. tabacum*) were grown under a 16 h photoperiod at 22 ± 2 °C. Abbreviations corresponding to samples are as follows: sepal (S), leaf (L), root (R), stem (S), opening flower (F), glandular trichomes (GTs), flower bud (FB). FB0, FB1, FB2, F3, F4, and F5 correspond to different stages of flower development, as described previously [13].

### RNA extraction and qPCR analysis

Total RNA was extracted from frozen samples using the HiPure Plant RNA Mini Kit (Magen, China) according to the manufacturer’s instructions. RNA quality and concentration were analyzed by gel electrophoresis and spectrophotometry. RNA was stored at − 80 °C until use. cDNA was synthesized according to the manufacturer’s instructions (Vazyme, China). Gene expression was measured by RT-qPCR on an Mx3000P system (Agilent Stratagene). Primers were designed using primer-BLAST (https://www.ncbi.nlm.nih.gov/ tools/primer-blast) (Additional file 15: Table S6). PCR and data analyses were performed as described previously [56].

### *LaMYC4* cloning and sequence analysis

Primers were designed based on the *LaMYC4* sequence obtained from the lavender genome (PRJNA642976) [37] (Additional file 15: Table S6), and the gene was amplified by PCR. The PCR product was cloned into the pBM16K vector and sequenced by TsingKe (Tianjin, China). Amino acid sequences homologous to LaMYC4 were retrieved from the NCBI database. Phylogenetic analysis was performed in MEGA software version 7.0 using the neighbor-joining method. Full length amino acid sequences of *Arabidopsis* bHLH proteins (AtbHLHs) were downloaded from the TAIR database (http:// www.arabidopsis.org). The reliability of the neighbor-joining tree was estimated by bootstrap analysis using 1000 bootstrap replications. The properties of the deduced amino acid sequence were predicted using ExPASy (http://web.expasy.org/compute_pi/).

### Subcellular localization and the transactivating activity of LaMYC4

The full-length cDNA of LaMYC4 was cloned using primers (Additional file 15: Table S6) containing KpnI restriction sites and was ligated into the expression vector pCAMBIA2300 to produce a fusion protein (35S::LaMYC4-GFP). The empty vector (pCAMBIA2300) and the recombinant vector (35S::LaMYC4-GFP) were transformed into *Agrobacterium tumefaciens* GV3101 by heat shock. Four-week-old *N. benthamiana* plants were transformed with the 35S::LaMYC4-GFP vector or 35S::GFP vector, as described previously [57]. After 3 days of transformation, leaves were removed and analyzed on a confocal laser scanning microscope equipped with a standard filter set (Leica TCS SP5).

For the transactivation assay, the full-length cDNA of *LaMYC4* was cloned into the pGBKT7 vector containing EcoRI and BamHI restriction sites. The negative control (pGBKT7), positive control (pGBKT7-p53), and recombinant vector were expressed in the yeast strain AH109 following the manufacturer’s instructions.

### Plant transformation and identification of transgenic lines

Bacterial colonies containing the 35S::LaMYC4-GFP vector were selected and transformed into the *Arabidopsis* Col-0 cultivar using a floral dip method [58] or tobacco plants using the leaf disk method [59]. Plants containing the empty vector served as a control. Explants were incubated in a growth chamber at 23 °C under a 16 h light/8 h dark photoperiod. Primary transformants were selected on half-strength Murashige and Skoog medium containing 50 μg mL^− 1^ kanamycin, and the presence of the transgene was confirmed by PCR.

### Measurement of volatile terpenoid concentrations

The volatile compounds released from lavender, tobacco, and *Arabidopsis* plants were collected by SPME [28, 37]. Fresh sepals (10 mg), fresh leaves (100 mg) from lavender, and fresh flowers (2 g) from tobacco were placed into headspace vials and kept at 40 °C (lavender sepals and leaves) or 60 °C (tobacco flowers) for 40 min and exposure to a DVB/CAR/PDMS fiber for 20 min, followed by analyte desorption at 250 °C for 3 min. A total of 0.25 μg of 3-octanol was added to these samples as an internal standard. To measure the release of volatiles by *Arabidopsis* plants, the plants were placed in a 25 cm × 38 cm plastic bag (EasyOven) and incubated at 23 ± 2 °C via DVB/CAR/PDMS fiber for 3 h, followed by analyte desorption at 250 °C for 3 min. The relative concentration of the target compounds was determined using standard curves, which were generated by three repeats: y = 10-7x + 0.0024 and *R*^2^ = 0.92 (Additional file 9: Fig. S9).

GC-MS analysis was performed via splitless injection using an Agilent 7890B GC system and an Agilent Technologies 7000C Inert XL Mass Selective Detector equipped with an HP-5MS UI column (30 m × 0.25 mm × 0.25 μm; Agilent Technologies), as described previously [37].

Products were identified based on retention times and electron ionization mass spectra obtained from the National Institute of Standards Technology (NIST) Mass Spectral Library (NIST-14.0) and literature data [35, 60, 61].

### Trichome morphology and number

Samples were examined on a field-emission scanning electron microscope (Hitachi S-4800), and the number and size of stem trichomes from the fourth fully grown internode of each plant were determined.

### Measurement of the level of anthocyanins and endogenous hormones

Twelve plants from each line were selected for measuring plant growth and total anthocyanin concentration. Total anthocyanins in tobacco flowers (500 mg) were measured as described previously [28]. GA, ABA, IAA, ZR, and JA in tobacco leaf were measured by enzyme-linked immunosorbent assay (ELISA). Hormones were extracted and purified according to He [62] and quantified by ELISA based on Yang et al. [63].

### Statistical analysis

Statistical analysis was performed by one-way analysis of variance followed by independent-samples *t*-test or Fisher’s least-significant difference test using SPSS software version 17.0. Data were expressed as the mean ± standard deviation of at least three independent experiments. *P*-values smaller than 0.05 were considered statistically significant.

## Supplementary Information

**Additional file 1: Figure S1**. Contents of volatiles from the lavender with 8 mM MeJA. (**a**) the contents of β-myrcene, β-cis-ocimene and caryophyllene in lavender sepal. (**b**) the contents of β-myrcene, β-cis-ocimene and caryophyllene in lavender leaf. Values shown are mean ± SD of three replicates. All data are given as the means ± SD (*n* = 3), *p < 0.05; **p < 0.01; ***p < 0.001; Student’s *t* test.

**Additional file 2: Figure S2**. Multiple alignment of nucleotide and amino acid. (**a**) nucleotide sequence. (**b**) amino acid sequence.

**Additional file 3: Figure S3**. Evolutionary tree analysis (circle tree) and subfamily classifications of bHLHs proteins in LaMYC4 and *Arabidopsis thaliana*. The evolutionary tree was constructed using the Neighbour-Joining method with 1000 bootstrap replication.

**Additional file 4: Figure S4**. Contents of caryophyllene from the *Arabidopsis* plants. Wild type (WT), transformed by the empty vector pCAMBIA2300S (2300) and overexpressed *LaMYC4* gene (35S::LaMYC4) plants (#2, #7). The products were identified by comparison with compounds in the library NIST14 and reference standards. Values shown are mean ± SD of three replicates. Standard errors are indicated as vertical lines on the top of each bar and bars annotated with different letters were significantly different according to Fisher’s LSD test (*P* < 0.05) after ANOVA.

**Additional file 5: Figure S5**. Contents of caryophyllene from the tobacco floral. Wild type (WT), transformed by the empty vector pCAMBIA2300S (2300) and overexpressed *LaMYC4* gene (35S::LaMYC4) plants (#3, #5). The products were identified by comparison with compounds in the library NIST14 and reference standards. Values shown are mean ± SD of three replicates. Standard errors are indicated as vertical lines on the top of each bar and bars annotated with different letters were significantly different according to Fisher’s LSD test (*P* < 0.05) after ANOVA.

**Additional file 6: Figure S6**. Hormone contents from the tobacco leaves. Hormone contents were measures by Enzyme-linked immunosorbent assay (ELISA). WT, wild type; 2300, transformed by the empty vector pCAMBIA2300S; #3 and #5, *LaMYC4* transgenic lines. Values shown are mean ± SD of three replicates. Standard errors are indicated as vertical lines on the top of each bar and bars annotated with different letters were significantly different according to Fisher’s LSD test (*P* < 0.05) after ANOVA.

**Additional file 7: Figure S7**. Phenotypic analysis of *LaMYC4* transgenic tobacco. (**a**) Phenotypes of plant and flower in wild type (WT), transformed by the empty vector pCAMBIA2300S (2300) and *LaMYC4* transgenic lines (#3, #5). (**b**) Results of plant height. (c) Total anthocyanin content in tobacco flower. (**d, e, f**) Surfaces cell of wild-type (WT), empty vector pCAMBIA2300 (2300) and overexpression of *LaMYC4* transgenic plants on stem (35S::LaMYC4). (g) Results of surfaces cell length on stem. Values shown are mean ± SD of replicates. The plant height was twelve replicates, the total anthocyanin contents was three replicates and the cell length were one hundred replicates. Standard errors are indicated as vertical lines on the top of each bar and bars annotated with different letters were significantly different according to Fisher’s LSD test (*P* < 0.05) after ANOVA.

**Additional file 8: Figure S8**. Transcript analysis of genes related to diterpenes synthesis in the tobacco floral. The values shown are mean ± SD at least three replicates. Standard errors are indicated as vertical lines on the top of each bar, and bars annotated with different letters were significantly different according to Fisher’s LSD test (*P* < 0.05) after ANOVA.

**Additional file 9: Figure S9**. Standard curves and mass spectrum. (**a**) standard curves. (**b**) Mass spectrum of the product. (**c**) Mass spectrum of caryophyllene.

**Additional file 10: Table S1.** Volatile compounds from control or treated with methyl jasmonate (MeJA) of lavender.

**Additional file 11: Table S2.** Sequences of LaMYC4 and 22 MYCs from different plants.

**Additional file 12: Table S3.** Promoter sequence of LaMYC4 and Prediction element.

**Additional file 13: Table S4.** Sequences of HMGR, FPPS, DXS, DXR, GPPS and TPS21.

**Additional file 14: Table S5.** Predicted functions of the LaMYC4 with the function of their homologs verified in *Arabidopsis* by phylogenetic analysis.

**Additional file 15: Table S6.** Primers used in this study.

## Data Availability

The raw genome and transcriptome sequencing data reported in this paper have been deposited in the National Center for Biotechnology Information (NCBI) database under project number PRJNA642976. And the data and materials in the current study are available from the corresponding author on reasonable request.
