# Supplementary material for: The transcription factor LaMYC4 from lavender regulates volatile Terpenoid biosynthesis
Source: BMC Plant Biol. 2022 Jun 13;22:289. doi: 10.1186/s12870-022-03660-3 (PMC9190104; doi:10.1186/s12870-022-03660-3)
Supplement: Supplementary file 1 — Additional file 1: Figure S1. Contents of volatiles from the lavender with 8 mM MeJA. (a) the contents of β-myrcene, β-cis-ocimene and caryophyllene in lavender sepal. (b) the contents of β-myrcene, β-cis-ocimene and caryophyllene in lavender leaf. Values shown are mean ± SD of three replicates. All data are given as the means ± SD (n = 3), *p < 0.05; **p < 0.01; ***p < 0.001; Student’s t test. [file 12870_2022_3660_MOESM1_ESM.docx]

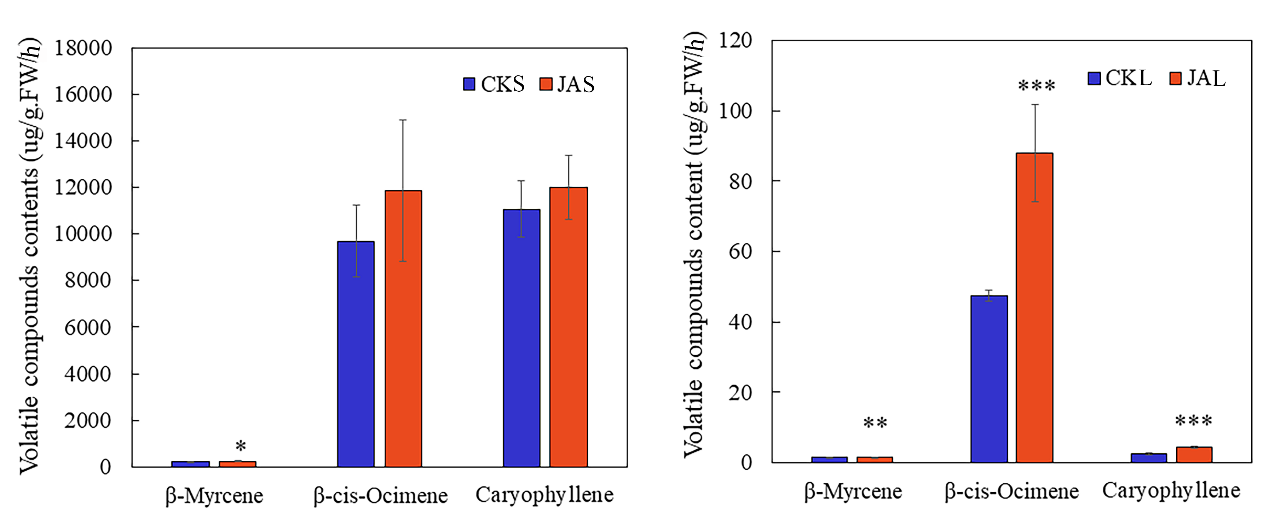


**Figure S1** Contents of volatiles from the lavender with 8 mM MeJA. (**a**) the contents of β-myrcene, β-cis-ocimene and caryophyllene in lavender sepals. (**b**) the contents of β-myrcene, β-cis-ocimene and caryophyllene in lavender leaves. Values shown are mean ± SD of three replicates. All data are given as the means ± SD (n = 3), **p* < 0.05; ***p* < 0.01; ****p* < 0.001; Student’s *t* test.
