# Supplementary material for: The transcription factor LaMYC4 from lavender regulates volatile Terpenoid biosynthesis
Source: BMC Plant Biol. 2022 Jun 13;22:289. doi: 10.1186/s12870-022-03660-3 (PMC9190104; doi:10.1186/s12870-022-03660-3)
Supplement: Supplementary file 2 — Additional file 2: Figure S2. Multiple alignment of nucleotide and amino acid. (a) nucleotide sequence. (b) amino acid sequence. [file 12870_2022_3660_MOESM2_ESM.docx]

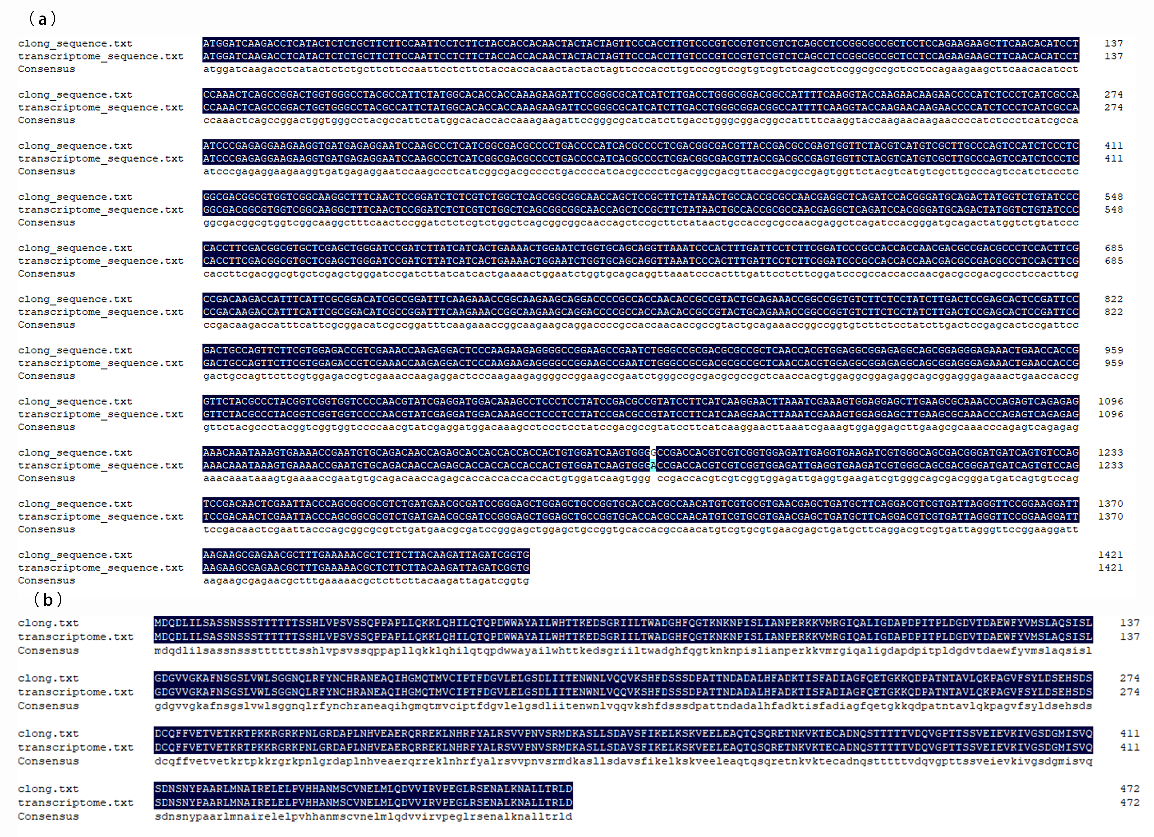


**Figure S2** Multiple alignment of nucleotide and amino acid. (**a**) nucleotide sequence. (**b**) amino acid sequence.
