# Supplementary material for: The transcription factor LaMYC4 from lavender regulates volatile Terpenoid biosynthesis
Source: BMC Plant Biol. 2022 Jun 13;22:289. doi: 10.1186/s12870-022-03660-3 (PMC9190104; doi:10.1186/s12870-022-03660-3)
Supplement: Supplementary file 3 — Additional file 3: Figure S3. Evolutionary tree analysis (circle tree) and subfamily classifications of bHLHs proteins in LaMYC4 and Arabidopsis thaliana. The evolutionary tree was constructed using the Neighbour-Joining method with 1000 bootstrap replication. [file 12870_2022_3660_MOESM3_ESM.docx]

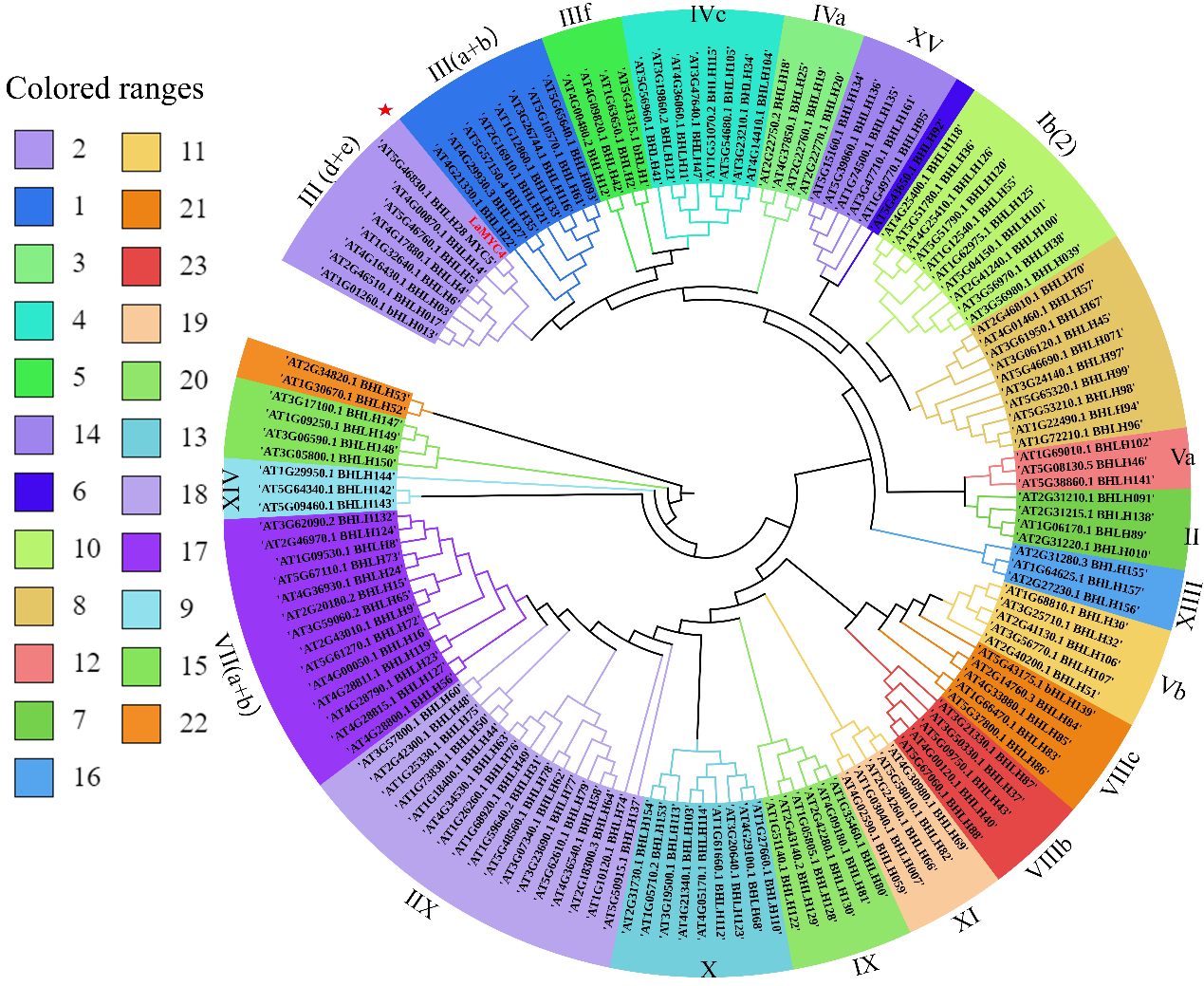


**Figure S3** Evolutionary tree analysis (circle tree) and subfamily classifications of bHLHs proteins in LaMYC4 and *Arabidopsis thaliana*. The evolutionary tree was constructed using the Neighbour-Joining method with 1000 bootstrap replication.
