# Supplementary material for: The transcription factor LaMYC4 from lavender regulates volatile Terpenoid biosynthesis
Source: BMC Plant Biol. 2022 Jun 13;22:289. doi: 10.1186/s12870-022-03660-3 (PMC9190104; doi:10.1186/s12870-022-03660-3)
Supplement: Supplementary file 4 — Additional file 4: Figure S4. Contents of caryophyllene from the Arabidopsis plants. Wild type (WT), transformed by the empty vector pCAMBIA2300S (2300) and overexpressed LaMYC4 gene (35S::LaMYC4) plants (#2, #7). The products were identified by comparison with compounds in the library NIST14 and reference standards. Values shown are mean ± SD of three replicates. Standard errors are indicated as vertical lines on the top of each bar and bars annotated with different letters were significantly different according to Fisher’s LSD test (P < 0.05) after ANOVA. [file 12870_2022_3660_MOESM4_ESM.docx]

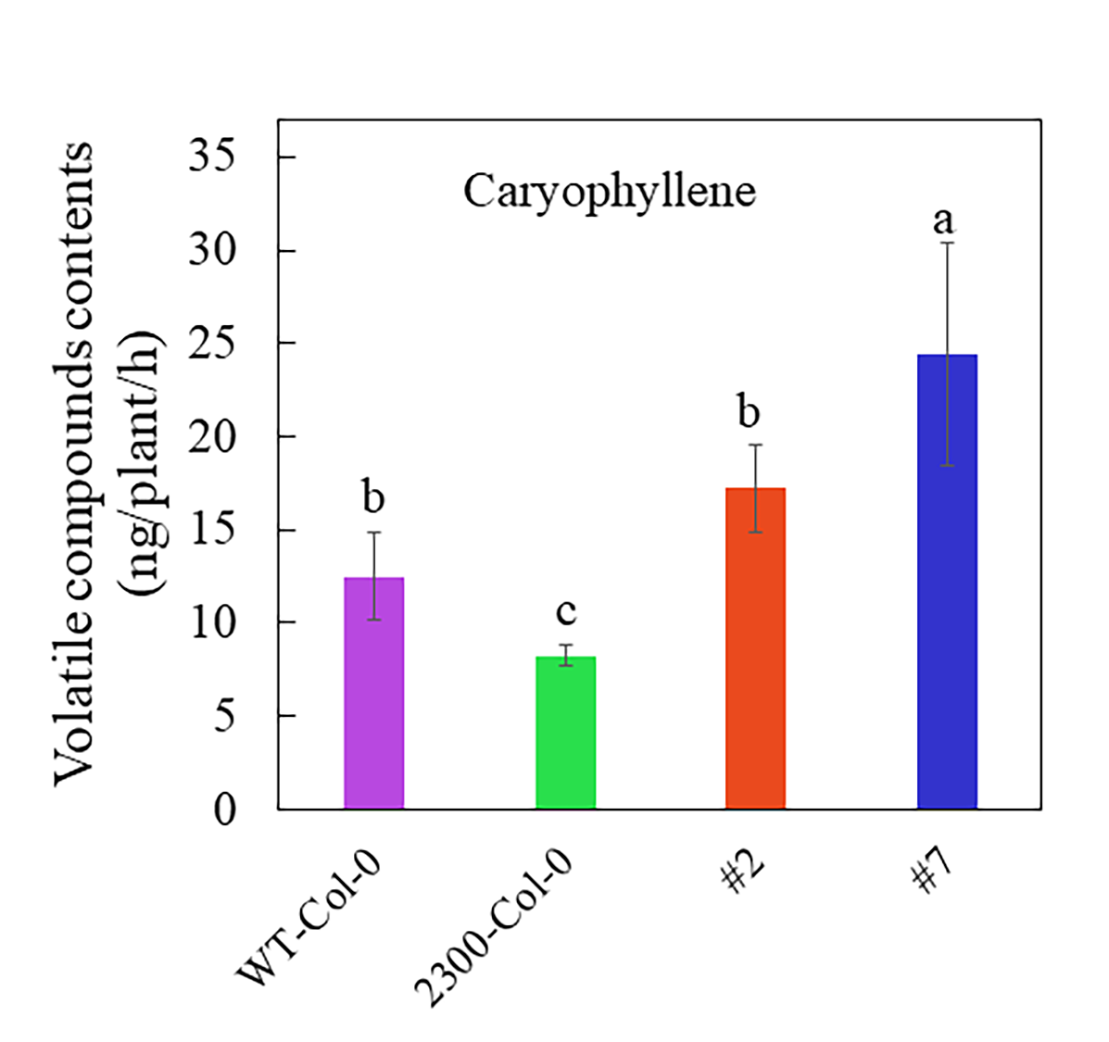


**Figure S4** Contents of caryophyllene from the *Arabidopsis* plants. wild type (WT), transformed by the empty vector pCAMBIA2300S (2300) and overexpressed *LaMYC4* gene (35S::LaMYC4) plants (#2, #7). The products were identified by comparison with compounds in the library NIST14 and reference standards. Values shown are mean ± SD of three replicates. Standard errors are indicated as vertical lines on the top of each bar and bars annotated with different letters were significantly different according to Fisher’s LSD test (*P* < 0.05) after ANOVA.
