# Supplementary material for: The transcription factor LaMYC4 from lavender regulates volatile Terpenoid biosynthesis
Source: BMC Plant Biol. 2022 Jun 13;22:289. doi: 10.1186/s12870-022-03660-3 (PMC9190104; doi:10.1186/s12870-022-03660-3)
Supplement: Supplementary file 6 — Additional file 6: Figure S6. Hormone contents from the tobacco leaves. Hormone contents were measures by Enzyme-linked immunosorbent assay (ELISA). WT, wild type; 2300, transformed by the empty vector pCAMBIA2300S; #3 and #5, LaMYC4 transgenic lines. Values shown are mean ± SD of three replicates. Standard errors are indicated as vertical lines on the top of each bar and bars annotated with different letters were significantly different according to Fisher’s LSD test (P < 0.05) after ANOVA. [file 12870_2022_3660_MOESM6_ESM.docx]

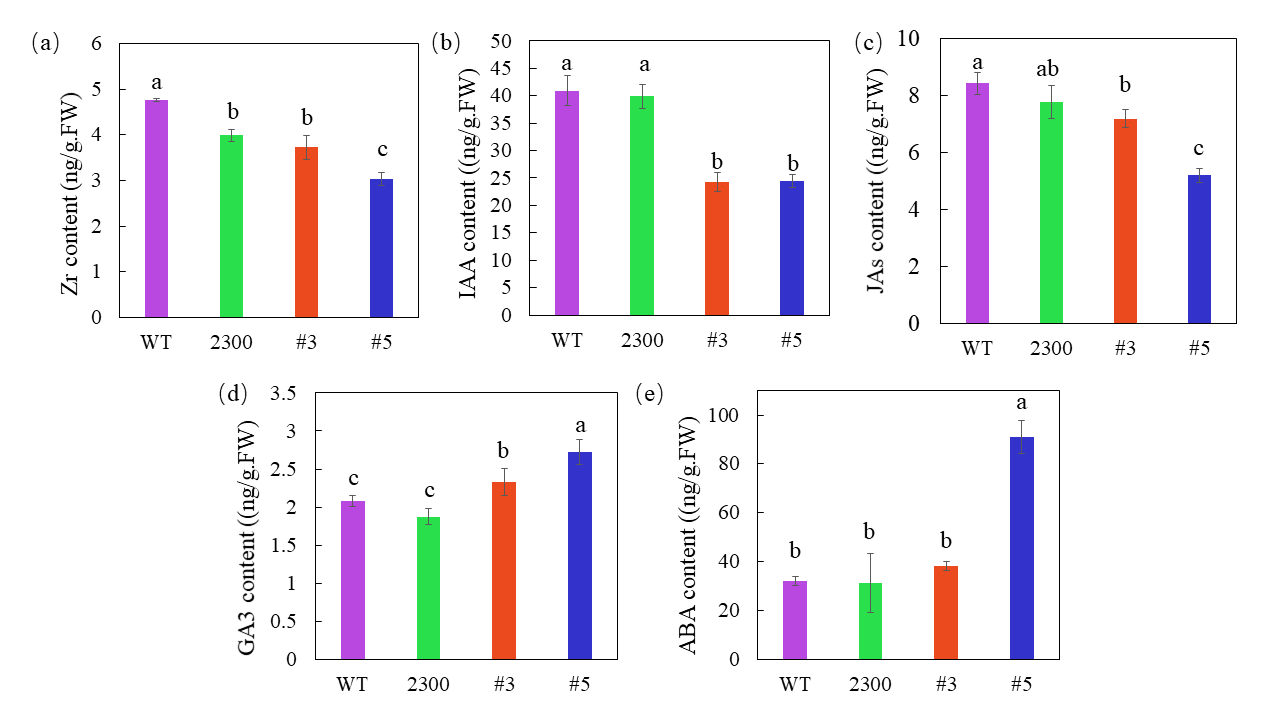


**Figure S6** Hormone contents from the tobacco leaves. Hormone contents were measures by Enzyme-linked immunosorbent assay (ELISA). WT, wild type; 2300, transformed by the empty vector pCAMBIA2300S; #3 and #5, *LaMYC4* transgenic lines. Values shown are mean ± SD of three replicates. Standard errors are indicated as vertical lines on the top of each bar and bars annotated with different letters were significantly different according to Fisher’s LSD test (*P* < 0.05) after ANOVA.
