# Supplementary material for: The transcription factor LaMYC4 from lavender regulates volatile Terpenoid biosynthesis
Source: BMC Plant Biol. 2022 Jun 13;22:289. doi: 10.1186/s12870-022-03660-3 (PMC9190104; doi:10.1186/s12870-022-03660-3)
Supplement: Supplementary file 7 — Additional file 7: Figure S7. Phenotypic analysis of LaMYC4 transgenic tobacco. (a) Phenotypes of plant and flower in wild type (WT), transformed by the empty vector pCAMBIA2300S (2300) and LaMYC4 transgenic lines (#3, #5). (b) Results of plant height. (c) Total anthocyanin content in tobacco flower. (d, e, f) Surfaces cell of wild-type (WT), empty vector pCAMBIA2300 (2300) and overexpression of LaMYC4 transgenic plants on stem (35S::LaMYC4). (g) Results of surfaces cell length on stem. Values shown are mean ± SD of replicates. The plant height was twelve replicates, the total anthocyanin contents was three replicates and the cell length were one hundred replicates. Standard errors are indicated as vertical lines on the top of each bar and bars annotated with different letters were significantly different according to Fisher’s LSD test (P < 0.05) after ANOVA. [file 12870_2022_3660_MOESM7_ESM.docx]

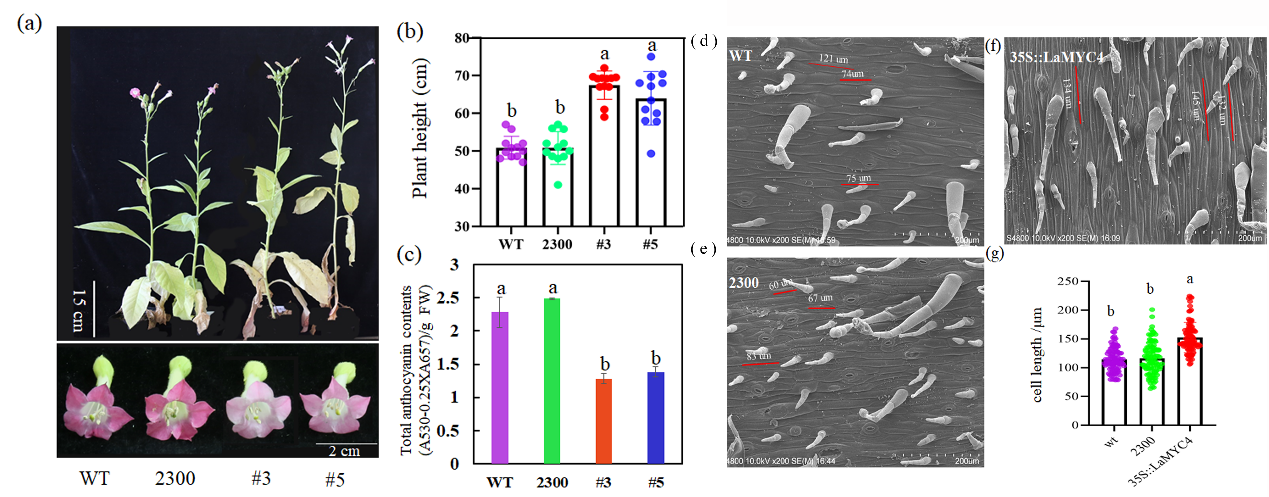


**Figure S7** Phenotypic analysis of *LaMYC4* transgenic tobacco. (**a**) Phenotypes of plant and flowers in wild type (WT), transformed by the empty vector pCAMBIA2300S (2300) and *LaMYC4* transgenic lines (#3, #5). (**b**) Results of plant height. (c) Total anthocyanin content in tobacco flowers. (**d, e, f**) Surfaces cell of wild-type (WT), empty vector pCAMBIA2300 (2300) and overexpression of *LaMYC4* transgenic plants on stem. (35S::LaMYC4). (g) Results of surfaces cell length on stem. Values shown are mean ± SD of replicates. The plant height was ten replicates, the total anthocyanin contents was three replicates and the cell length were one hundred replicates. Standard errors are indicated as vertical lines on the top of each bar and bars annotated with different letters were significantly different according to Fisher’s LSD test (*P* < 0.05) after ANOVA.
