# Supplementary material for: The transcription factor LaMYC4 from lavender regulates volatile Terpenoid biosynthesis
Source: BMC Plant Biol. 2022 Jun 13;22:289. doi: 10.1186/s12870-022-03660-3 (PMC9190104; doi:10.1186/s12870-022-03660-3)
Supplement: Supplementary file 8 — Additional file 8: Figure S8. Transcript analysis of genes related to diterpenes synthesis in the tobacco floral. The values shown are mean ± SD at least three replicates. Standard errors are indicated as vertical lines on the top of each bar, and bars annotated with different letters were significantly different according to Fisher’s LSD test (P < 0.05) after ANOVA. [file 12870_2022_3660_MOESM8_ESM.docx]

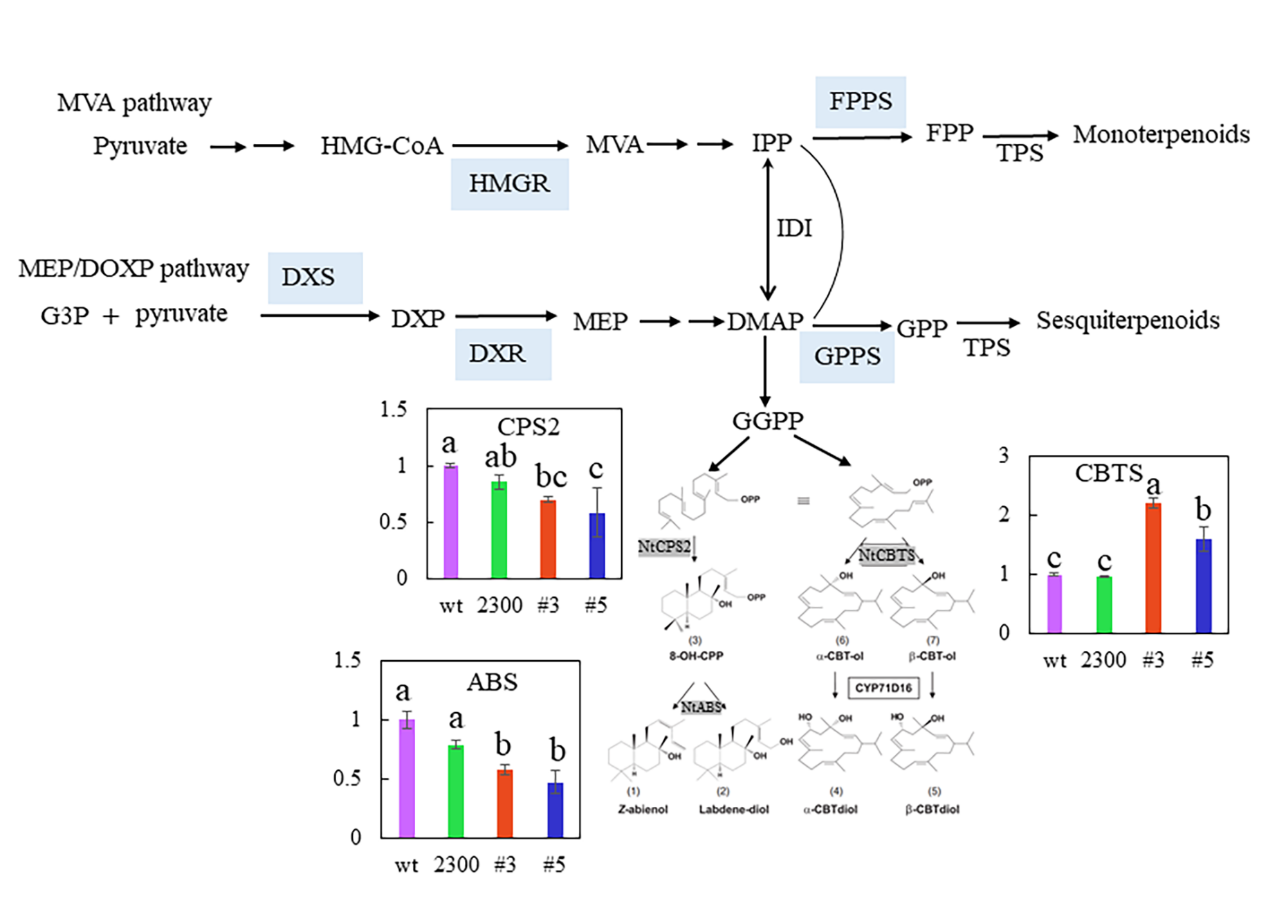


**Figure S8** Transcript analysis of genes related to diterpenes synthesis in the tobacco floral. The values shown are mean ± SD at least three replicates. Standard errors are indicated as vertical lines on the top of each bar, and bars annotated with different letters were significantly different according to Fisher’s LSD test (*P* < 0.05) after ANOVA.
