# Supplementary material for: The transcription factor LaMYC4 from lavender regulates volatile Terpenoid biosynthesis
Source: BMC Plant Biol. 2022 Jun 13;22:289. doi: 10.1186/s12870-022-03660-3 (PMC9190104; doi:10.1186/s12870-022-03660-3)
Supplement: Supplementary file 9 — Additional file 9: Figure S9. Standard curves and mass spectrum. (a) standard curves. (b) Mass spectrum of the product. (c) Mass spectrum of caryophyllene. [file 12870_2022_3660_MOESM9_ESM.docx]

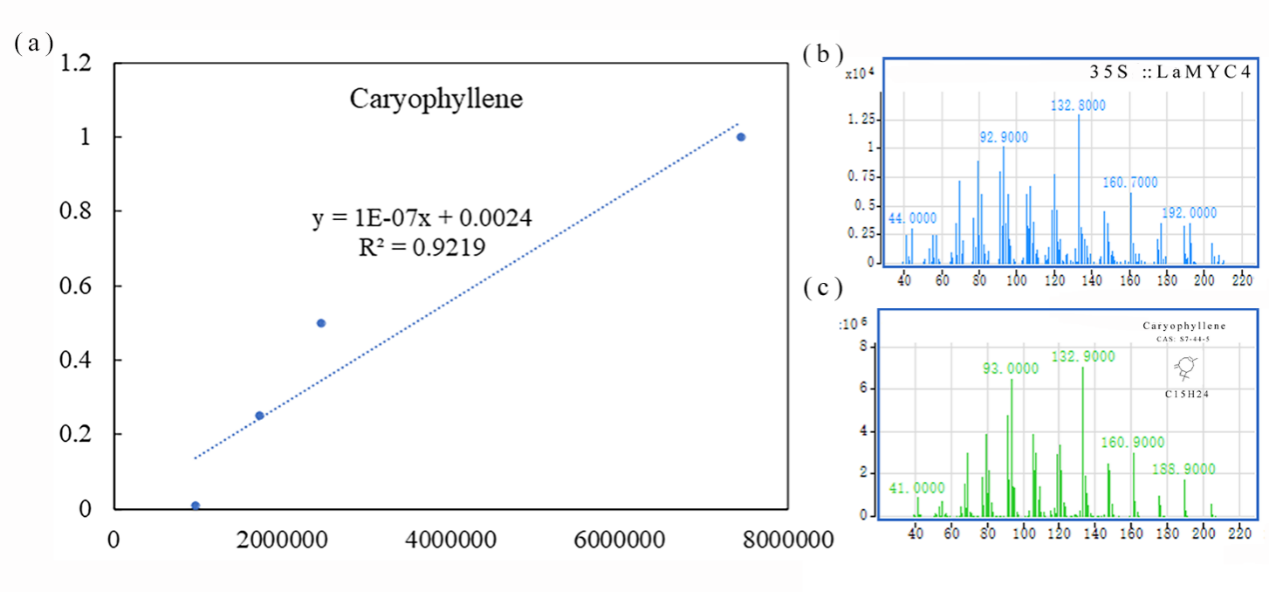


**Figure S9** standard curves and mass spectrum. (**a**) standard curves. (**b**) Mass spectrum of the product. (**c**) Mass spectrum of caryophyllene.
