# Supplementary material for: The transcription factor LaMYC4 from lavender regulates volatile Terpenoid biosynthesis
Source: BMC Plant Biol. 2022 Jun 13;22:289. doi: 10.1186/s12870-022-03660-3 (PMC9190104; doi:10.1186/s12870-022-03660-3)
Supplement: Supplementary file 15 — Additional file 15: Table S6. Primers used in this study. [file 12870_2022_3660_MOESM15_ESM.docx]

**Table S1** Primers used in this study.

| Gene Name | Primer name | Primer sequences (5′→3′) | Purposes |
| --- | --- | --- | --- |
| LaMYC4-2300 | Forward | TCGATACACCAAATCGACTCTAGAAA | ligated to the pCAMBIA2300 vector |
|  |  | ATGGATCAAGACCTCATACTCTCTG |  |
|  | Reverse | CCCCGGGCCCCTGCAGAAGC |  |
|  |  | CCGATCTAATCTTGTAAGAAGAGC |  |
| LaMYC4-BK | Forward | GCATATGGCCATGGAGGCCG | ligated to the pGBKT7 vector |
|  |  | AATTC ATGGATCAAGACCTCATACTC |  |
|  | Reverse | CGGCCGCTGCAGGTCGAC |  |
|  |  | GGATCC TCACCGATCTAATCTTGTAAG |  |
| qpcr-LaMYC4 | Forward | GTGGTCCCCAACGTATCGAG | qPCR quantification |
|  | Reverse | TCTCTGACTCTGGGTTTGCG |  |
| qPCR-HMGR | Forward | GCAGGTGGCGTGAGAAAATC | qPCR quantification |
|  | Reverse | CCCTGGAAACGAACGACTGA |  |
| qPCR-DXS | Forward | CACCGAGAAAGGCAGAGGTT | qPCR quantification |
|  | Reverse | CCAACATCAAAACACCGCGT |  |
| qPCR-GGPS | Forward | GTTGCTGATAAGGTAACTTATCCCAAAC | qPCR quantification |
|  | Reverse | CAGATCACTCATGTTTTCACCTTCTATT |  |
| qPCR-FPPS | Forward | GTACTCATTGCCTATTCATCG | qPCR quantification |
|  | Reverse | CACTACCAACCAAGAGCAC |  |
| qPCR-DXR | Forward | CTCTACACGGCTGTCCCTAACT | qPCR quantification |
|  | Reverse | GGTGTCCAAGAAAGAGATGGTC |  |
| qPCR-TPS21 | Forward | GTCCACTAGCTGATTTTCCA | qPCR quantification |
|  | Reverse | TCTCTATCGGATTCGCTTTG |  |
| qPCR-Tubulin | Forward | TCAAGAGGTTCTCAGCAGTA | qPCR quantification |
|  | Reverse | TCACCTTCTTCATCCGCAGTT |  |
| qPCR-β-actin | Forward | GGTAGCTCCACCTGAGAGGAAGT | qPCR quantification |
|  | Reverse | GCCTTTGCAATCCACATCTGT |  |
| La18 s rRNA | Forward | GTG ACG GGT GACGGA GAA | qPCR quantification |
|  | Reverse | GAC TCA ATG AGC CCG GTA |  |
| Laβ-actin | Forward | TGT GGA TTG CCA AGG CAG AGT | qPCR quantification |
|  | Reverse | AAT GAG CAG GCAGCAACA GCA |  |
| qPCR-Tub | Forward | CAACCTCTGTGGTGGAACCT | qPCR quantification |
|  | Reverse | GGAACAAGGTTGGTCTGGAA |  |
| qPCR-CBTS | Forward | CGACTTGCGAGGCAACAAGG | qPCR quantification |
|  | Reverse | CGACTTGCGAGGCAACAAGG |  |
| qPCR-CPS2 | Forward | TGGACCCCGAAATTGGTTGT | qPCR quantification |
|  | Reverse | ACCCAAGCTGTGTCATAGGC |  |
| qPCR-ABS | Forward | GTGCCAAACCCGACACAAAG | qPCR quantification |
|  | Reverse | ACGGTTGAGTATCCCTCCCA |  |
| qPCR-CYP | Forward | TTCCTCTCCATGGCTAATGC | qPCR quantification |
|  | Reverse | ATCCAGATCCAACAGCCTCA |  |
